# Editorial: Emerging Solidarities on the Ground in the Management and Approach of the COVID-19 Emergency

**DOI:** 10.3389/fsoc.2021.693482

**Published:** 2021-05-04

**Authors:** Emilia Aiello, Elias Nazareno

**Affiliations:** ^1^ Harvard University, Kenndey School of Government, MA, United States; ^2^ Facultad de Historia y Licenciatura en Educación Intercultural IndÍgen, Universidad Federal de Goias, Goiânia, Brazil

**Keywords:** COVID19, grassroots actors, public leadership, at-risk communities, inequalities

The manuscripts published in the Special Issue *Emerging Solidarities on the Ground in the Management and Approach of the COVID emergency* made it possible to approach the different coping strategies and management of the COVID 19 pandemic in different parts of the world, reveals the enormous human capacity to respond to problems as challenging as the pandemic was and still is. Whether in large European cities or within the Brazilian Amazon, in different forms of social organization, it was possible to perceive an enormous capacity for resistance and resilience in the face of a problem of incalculable proportions and damage.

This Special Issue gathers eight different papers, from authors representing Latin American institutions, and Canadian and European.

The first manuscript, *The Covid-19 Emergency and the Risk of Social Fragmentation in the Palermo case,* by Siino, the experiences of associations working with people belonging to the weakest part of the community were analyzed from the social context in Southern Italy, in Palermo. Siino interviewed representatives of the main local associations as privileged witnesses, highlighting how the activities of local actors and how the dynamics of solidarity are influenced by the global Covid-19 phenomenon.

Also referring to Italy, the *manuscript The Italian Deaf community at the time of Coronavirus*, by Tomasuolo et al., presented an analysis of the impacts of the pandemic crisis on the Italian deaf community as a linguistic minority. Authors also analyzed, how social media were exploited as a basis to promote social cohesion and share information about the emergence of the coronavirus, and how the deaf community shaped interpretation services in the public media. Hence, the use of social media allowed Deaf people to create a new virtual space.

Moving to a different geographical context, the manuscript *Covid-19 and the Brazilian Reality: The Role of Favelas in Combating the Pandemic* by Carvalho Fernandes et al., showed that favelas’ inhabitants are victims of the enormous social inequalities present in Brazilian society and that these are even more severe in the slums. Favelas suffer from lack of access to decent housing, potable water, and a minimum income for survival, what makes the effects of the pandemic more devastating. However, the organizing of actors on the ground and the creation of “Crisis Offices” in the slums led by social organizations and support institutions, has made possible to facilitate mechanisms for assistance.

Three manuscripts in this Special Issue tackle the issue of COVID-19 and how it has impacted on the Brazilian indigenous communities, either deepening on how these communities have taken an active role organizing to put up actions to serve their communities in a moment where the government was not succeeding to do so, or reflecting on how these communities have put up indigenous knowledge to shape these strategies and make them more effectives.

First, in *Afro-Indigenous Cosmographies of Mobility: Fishes, Viruses and Other Amazonian Lives at the Confluence With the Sars-CoV-19,* Baines et al., depict the susceptibility to death that certain Amazonian peoples are facing as a result of their particular migratory demography which, instead of being contained, has been exponentially intensified by the outbreak of COVID-19. The article demonstrates how the cosmographies of mobility of these populations are being challenged by the changes and strategic conditions imposed by the pandemic.

Second, the manuscript *The Articulation of the Indigenous Peoples of Brazil in Facing the Covid-19 Pandemic,* da Silva et al., analyzed how the indigenous communities of Brazil have organized autonomous actions and strategies to confront the COVID-19 pandemic. What is interesting for this case is that in articulating these actions and strategies, indigenous communities have brought in their lifeworlds and their own historical experiences, their health conceptions, partnerships with scientific communities and other segments of society that support the indigenous struggle.

And third, the paper *Urgent considerations on the relationship between the advance of Covid-19 in indigenous territories in Brazil and the impacts of monoepistemic public policies*, authored by Herbetta et al., analyzed the advance of Covid-19 in indigenous territories in Brazil, whether urban or rural. In this paper authors do a theoretical discussion and from it, they observe that policies need to change their old paradigms, which most of the time are centered on the fragmentation of knowledge, the rationalization of the world and on cultural and human distance. Herbetta et al. suggest that public policies and state institutions should be anchored in co-theoretical and intercultural foundations, incorporating different languages and forms of expression in the dialogical process of managing demands.

Also in Brazil but from a broader angle and beyond the scope of indigenous communities, Arrais et al., analyze in the manuscript *The Role Played by Public Universities in Mitigating the Coronavirus Catastrophe in Brazil: Solidarity, Research and Support to Local Governments Facing the Health Crisis* the role played by higher education institutions (universities) in Brazil and how they have partnered with the government and other civil society organizations to face COVID-19. Arrais and colleagues show that through specific university initiatives such as the issuing of alerts to society on the risks of the pandemic, direct assistance to local communities, with emphases on the addition of beds in university hospitals for treating patients with COVID-19 and on the manufacturing of personal protective equipment, and research to find solutions to prevent and treat the disease, Brazilian public Federal Universities area also playing a role in supporting both civil society and local governments in mitigating the impacts of the pandemic. It is worth paying attention as how higher education institutions are partnering and seeking allies with other institutions operating at the grassroots level, as well as with local and regional governments to seek common ground to help keep the population informed.

And finally, the manuscript *Practicing social isolation during a pandemic in Brazil: a description of psychological characteristics and traits of personality during COVID-19 lockdown*, a collaboration among authors from Brazil and Canada, Zanini et al., explores the importance of considering the psychological characteristics and their influence on the social behavior of individuals and on supportive (pro-social) behavior, even in a period of significant stress and high social risk, and how this should be much taken into account at the time of design and implement public policies. The results showed that respondents practicing social isolation to comply with governmental recommendations had lower scores on the scales of neuroticism and consciousness; they reported less stress, anxiety, and depression, and less general distress.

We hope these cases can shed some light on how diverse societal actors operating at the local level and well-organized are coordinating efforts worldwide and organizing to alleviate the impact, especially on those most at risk communities. We have been for one year now in this pandemic, and what authors explain in these articles is the results of the fast response mostly at the community level, in a moment where we were all trying to making sense of what was occurring. Hopefully, these investigations will serve to set the ground for future collaborations and ideas on resilience and cooperation, as well as the urgent gaps that still are needed to be covered.

